# *TCF7L1* Genetic Variants Are Associated with the Susceptibility to Cervical Cancer in a Chinese Population

**DOI:** 10.1155/2021/6670456

**Published:** 2021-03-20

**Authors:** Jingjing Chen, Yuanfang Xu, Hongyuan Hu, Tianbo Jin

**Affiliations:** ^1^Gynecology Department, People's Hospital of Wanning, Wanning, 571500 Hainan, China; ^2^Obstetrical Department, People's Hospital of Wanning, Wanning, 571500 Hainan, China; ^3^Key Laboratory of Molecular Mechanism and Intervention Research for Plateau Diseases of Tibet Autonomous Region, School of Medicine, Xizang Minzu University, Xianyang, 712082 Shaanxi, China; ^4^Key Laboratory of Resource Biology and Biotechnology in Western China, Ministry of Education, Northwest University, Xi'an, 710069 Shaanxi, China

## Abstract

**Background:**

Cervical cancer (CC) is the second most common tumor in women worldwide. Studies have been accepted that genetic variations play an important role in the development of CC. The aim of this study was to evaluate the impact of *TCF7L1* variants on CC risk.

**Methods:**

508 patients of cervical cancer and 497 healthy subjects were recruited to determine the impact of *TCF7L1* polymorphisms on CC susceptibility. The associations were investigated by computing odds ratios (ORs) and 95% confidence intervals. The effect of SNP-SNP interactions on CC risk was explored by multifactor dimensionality reduction analysis.

**Results:**

Our study showed that rs11904127 (OR 0.79, *p* = 0.010) and rs62162674 (OR 0.82, *p* = 0.044) of *TCF7L1* significantly decreased cervical cancer risk. Stratified analysis indicated that rs11904127 and rs62162674 present decreased susceptibility to CC in age > 51 years (OR 0.74, *p* = 0.019; OR 0.72, *p* = 0.014, respectively). Haplotype analyses revealed that G_rs2366264_T_rs11689667_C_rs62162674_ has a lower risk to cervical cancer (OR = 0.43, *p* = 0.018). Besides, there is strong interaction of rs11904127 and rs2366264.

**Conclusion:**

Rs11904127 and rs62162674 in *TCF7L1* are related to cervical cancer. We suggest that these variants can be used as prognostic markers for judging the susceptibility to cervical cancer.

## 1. Introduction

Cervical cancer (CC) is the second most common tumor in women worldwide [1]. A survey in 2018 reported that there have approximately 311,000 deaths and 570,000 new cases [2]. Although infection with human papillomavirus (HPV) has been recognized as a vital risk factor for CC [3], HPV infection alone is not enough to lead to the occurrence of CC [4, 5]. The pathogenesis and susceptibility of CC are linked with the HPV infection-host factor interaction. For the host factor, the genetic variation of susceptible loci may have a significant impact on the risk of CC [6]. Growing studies have demonstrated that genetic variants also markedly contribute to the initiation and progression of CC [7–9]. Additionally, numerous researches have shown that genetic variants such as *XRCC1*, *OGG1*, *IL-6*, *MTHFR*, and *ERCC1* are associated with the risk of CC [10–12]. The study of genetics has increased our understanding of the pathogenesis of CC, so it may be very important to identify genetic risk factors for CC.

The transcription factor 7 like 1 (TCF7L1, also known as TCF3) is a member of the lymphoid enhancer/T cytokine transcription factor family. *TCF7L1* can regulate the Wnt target genes' expression by interacting with *β*-catenin which is a regulator of the Wnt signaling pathway and acts as a DNA-specific binding protein [13]. In different kinds of human cancer, it has been found that the *TCF7L1* gene is abnormally expressed in tumor tissues and has tumor regulatory function. Silencing of *TCF7L1* can significantly inhibit the growth of cancer cells, while overexpression of *TCF3* can promote cancer cell proliferation in prostate cancer [14]. Besides, the expression of *TCF7L1* was found to be upregulated in high malignant tumors and was associated with poor survival. For instance, *TCF7L1* positively regulates cell proliferation in colorectal cancer, and silencing its expression can reduce the size of xenografted tumors [15]. In leukemia, the knockdown of *TCF7L1* could reduce tumor growth [16]. Nowadays, increasing studies revealed that *TCF7L1* is an essential factor in the occurrence and progression of cervical cancer. Luo et al. showed that the high-level expression of *TCF7L1* can significantly accelerate the proliferation, invasion, and migration of cervical cells and negatively related to the prognosis of cervical squamous cell carcinoma (CSCC) [17]. Another study also indicated that the downregulation of *TCF7L1* has an obvious antitumor effect on cervical cancer [18]. As we know, single nucleotide polymorphisms (SNPs) may affect the expression level of genes or the function of proteins, which can help us to predict the susceptibility to many diseases or to study the role of genetic factors in disease progression [19]. Above all, we speculated that polymorphisms in *TCF7L1* have an important role in the progression of CC. To our knowledge, there is no report about the polymorphisms associated with the risk of CC.

In the current study, four SNPs (rs11904127, rs2366264, rs11689667, and rs62162674) in *TCF7L1* were obtained from 1000 genomes project and genotyped by MassARRAY platform. We then explored the relationship between TCF7L1 variations and cervical cancer in a Chinese population. We also detected the association stratified by age. Finally, MDR was carried out to investigate the impact of SNP-SNP interactions on CC susceptibility. Our study will provide a new genetic risk factor for the identification of CC in a Chinese population.

## 2. Materials and Methods

### 2.1. Study Population

A total of 1005 unrelated Chinese women included 508 patients with cervical cancer, and 497 healthy individuals were recruited in this case-control study at Shaanxi Provincial Cancer Hospital. Before starting the research, we informed all participants of the study purpose and everyone signed a written informed consent form. Patients were newly diagnosed and confirmed to be cervical cancer according to histological diagnosis. The patients who had a history of other cancers, chemotherapy, radiotherapy, or chemoradiotherapy treatment, family history of cervical cancer, cardiovascular disease, and infection were excluded. Control groups were selected from the healthy subjects who have normal physical examination at the same time. The healthy controls had no family history of cancers and have a normal cervix in accordance with histological examination. The clinical data of each individual were summarized from medical records including age, tumor type, and clinical stage. Our study was approved by the Ethics Committee of Shaanxi Provincial Cancer Hospital, and all experiments are performed under the protocol of Helsinki's Declaration.

#### 2.1.1. Selection and Genotyping of *TCF7L1* Polymorphisms

The selection of the *TCF7L1* SNPs must meet the following criteria. First, the candidate SNPs with minor allele frequencies > 5% on the basis of the 1000 Genome Project. Second, the call rate of each SNP was greater than 95%. Third, the genotype distributions of the SNPs in control groups were in accordance with Hardy-Weinberg equilibrium (HWE) (*p* > 0.05). Finally, four SNPs including rs11904127, rs2366264, rs11689667, and rs62162674 were selected from the 1000 Genomes Project database for the Chinese Han Beijing (CHB) population. A DNA extraction kit (GoldMag Co. Ltd., Xi'an, China) was used to extract genomic DNA from peripheral blood samples. Primers for amplification in the current study were designed by Agena Design software, and primer sequences were present in Table 1. The SNP genotyping was tested by the Agena MassARRAY iPLEX platform (Agena Bioscience Inc., CA, USA). The PCR reaction consisted of 1 *μ*L of 10 ng/*μ*L genomic DNA and 4 *μ*L of PCR mixture that contained 1.8 *μ*L of water, 0.5 *μ*L of 10× PCR buffer, 0.4 *μ*L of 25 mM MgCl_2_, 0.1 *μ*L of 25 mM dNTP, 1 *μ*L of PCR Primer mix, and 0.2 *μ*L of 5 U/*μ*L PCR Taq. The PCR conditions were as follows: initial denaturing at 95°C for 2 min, followed by 45 cycles of denaturing at 95°C for 30 s, annealing at 56°C for 30 s, and final extension at 72°C for 60 s. Then, the final step is to keep it at 25°C indefinitely. Matrix-assisted laser desorption/ionization-time of flight (MALDI-TOF) mass spectrometry was used to identify SNP alleles of different quality extension primers after alkaline phosphatase reaction, single group extension, and resin desalination reaction. We finally organized and analyzed the data of genotyping by using Agena Bioscience TYPER version 4.0 software. The representative spectra of each SNP were shown in Figure S1.

### 2.2. Statistical Analyses

All statistical analysis of this study was tested by the SPSS software (version 17.0). The *p* value was calculated by statistical tests with two-tailed. *p* < 0.05 indicates statistical significance. Differences in age and clinical indicators between the cases and controls were compared by student's *t*-test. HWE (Hardy-Weinberg equilibrium) in the control group was measured by Fisher's exact test. The correlation between *TCF7L1* variations and cervical cancer risk was detected using logistic regression analysis with adjustment for age under allele, dominant, recessive, codominant, and log-additive models. We further determined the association stratified by age. ORs and 95% CI (confidence intervals) were used to study the associations. We further constructed Linkage disequilibrium (LD) using Haploview software. Haplotype analysis was determined by logistic regression analysis. We finally explored the SNP-SNP interactions in the susceptibility of cervical cancer by using a multifaceted dimensionality reduction (MDR) method.

## 3. Results

### 3.1. Basic Information on the Study Population

As is presented in Table 2, this study consisted of 508 cervical cancer patients and 497 healthy subjects. The mean age was 51.62 ± 9.78 years in the cases and 51.36 ± 10.30 years in the controls. There is no statistical difference in age between the case and the control group (*p* = 0.684).

### 3.2. The Impact of *TCF7L1* Variants on Cervical Cancer Risk

In this case-control study, four SNPs (rs11904127, rs2366264, rs11689667, and rs62162674) were successfully genotyped. The allele frequencies for SNPs of the *TCF7L1* gene were summarized in Table 3. All SNPs in the control group were following HWE (all *p* > 0.05). The position of the SNPs in the *TCF7L1* gene was shown in Figure S1. The correlation between *TCF7L1* variants and cervical cancer risk was examined by the multiple genetic models with adjustment for age. Our result showed that two SNPs are significantly related to the risk of cervical cancer (Table 4). The rs11904127 of *TCF7L1* significantly decreased cervical cancer risk under allele model (A vs. G, OR 0.79, *p* = 0.010), codominant model (GA vs. GG, OR 0.68, *p* = 0.006; AA vs. GG, OR 0.67, *p* = 0.043), dominant model (GA-AA vs. GG, OR 0.68, *p* = 0.003), and Log-additive model (OR 0.79, *p* = 0.010). Rs62162674 also played a protective role in the cervical cancer under allele model (C vs. G, OR 0.82, *p* = 0.044) and Log-additive model (OR 0.81, *p* = 0.038).

### 3.3. Stratification Analyses for the Relationship of *TCF7L1* Variants with Cervical Cancer Risk

We further evaluated the association of SNPs with cervical cancer risk stratified by age, and the data is shown in Table 5. In age > 51 years, we observed that rs11904127 was significantly associated with a decreased risk of cervical cancer under allele model (A vs. G, OR 0.74, *p* = 0.019), codominant model (GA vs. GG, OR 0.67, *p* = 0.049; AA vs. GG, OR 0.58, *p* = 0.040), dominant model (GA-AA vs. GG, OR 0.65, *p* = 0.021), and Log-additive model (OR 0.74, *p* = 0.021). Rs62162674 showed a protective role in cervical cancer risk under allele model (C vs. G, OR 0.72, *p* = 0.014), codominant model (CC vs. GG, OR 0.50, *p* = 0.028), dominant model (GA-AA vs. GG, OR 0.67, *p* = 0.028), and Log-additive model (OR 0.71, *p* = 0.014).

### 3.4. Haplotype Analyses of *TCF7L1* Variants and Cervical Cancer Risk

**Figure 1:**
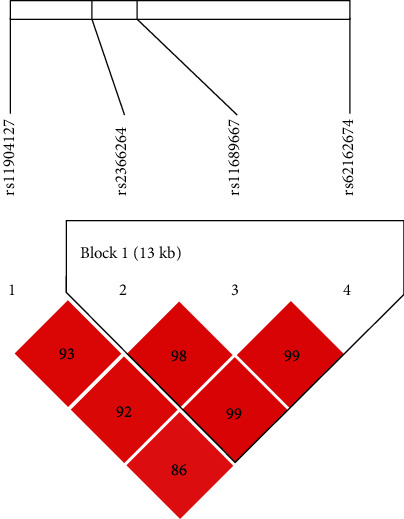
Haplotype block map for SNPs in the *TCF7L1* gene. The numbers inside the diamonds indicate the D′ for pairwise analyses.

We also determined LD and haplotype analyses of the *TCF7L1* SNPs. LD was constructed and presented in Figure 1; there were three SNPs including rs2366264, rs11689667, and rs62162674 forming a block. The frequency of haplotypes in the cases and controls were summarized in Table 6. The haplotype analyses showed that G_rs2366264_T_rs11689667_C_rs62162674_ has a lower susceptibility to cervical cancer (OR 0.43, *p* = 0.018).

### 3.5. Impact of SNP-SNP Interaction on Cervical Cancer Risk

**Figure 2:**
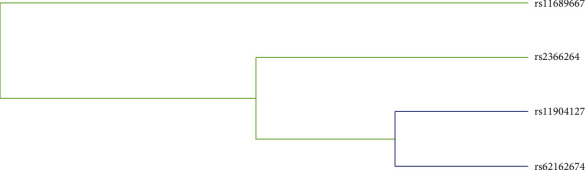
SNP-SNP interaction dendrogram. Yellow and blue represent redundancy or correlation.

We finally used the MDR analysis to test the influence of SNP-SNP interaction. As shown in Figure 2, the interactions between these SNPs were detected by the dendrogram. Interestingly, there are strong interactions between rs11904127 and rs2366264. As presented in Table 7, we observed that rs11904127 was the best single-locus model to predict cervical cancer (testing accuracy 0.540, CVC 10/10, *p* = 0.001). The best two-locus model was consisted of rs11904127 and rs2366264 (testing accuracy 0.507, CVC 7/10, *p* = 0.001). The best three-locus model was the combination of rs11904127, rs2366264, and rs62162674 (testing accuracy 0.507, CVC 10/10, *p* < 0.0001). The combination of rs11904127, rs2366264, rs11689667, and rs62162674 was the best four-locus model (testing accuracy 0.499, CVC 10/10, *p* < 0.0001).

## 4. Discussion

In this study, we firstly detected the impact of *TCF7L1* SNPs on cervical cancer risk in a Chinese population. Our study indicated that rs11904127 and rs62162674 were significantly decreased cervical cancer risk. Besides, we found that rs11904127 and rs2366264 polymorphism have a strong interaction in the aspect of cervical cancer risk. These data suggest that *TCF7L1* genetic variations have a strong association with cervical cancer.

Cervical cancer is a complex disease and remains a serious health problem in both developed and developing countries. Previous epidemiological studies showed that cervical cancer is mainly caused by high-risk HPV infection [3, 20]. At present, it is generally believed that genetic factors also act as a crucial role in pathogenesis [21, 22]. Besides, increasing studies reported that genetic polymorphisms are linked with cervical cancer risk [23–25]. The *TCF7L1* gene is located on chromosome 2p11.2 and is a protein-coding gene. *TCF7L1* is involved in the occurrence and progression of many human tumors such as endometrial cancer, breast cancer, and gastric cancer [26, 27]. Recent evidence indicated that abnormal expression of the TCF7L1 gene contributed to the occurrence of cervical cancer by regulating tumor behaviors including cell growth, invasion, and migration [17, 18]. *TCF7L1* genetic variants may relate to cervical cancer progression according to the impact of the gene expression level. To our knowledge, there has no study exploring the correlation between *TCF7L1* genetic variants and cervical cancer risk. We tried to detect the association, and we found that rs11904127 and rs62162674 showed a protective effect on cervical cancer. When stratified by age, rs11904127 and rs62162674 polymorphisms significantly decreased the susceptibility to cervical cancer in age > 51 years. No significant association was observed in age ≤ 51 years. We guess that age may be a potential factor in cervical cancer susceptibility.

By detecting the interaction of SNP-SNP, we can find the risk factors of gene-environment interaction on the pathogenesis of cervical cancer. Our result presented that there are strong interactions between rs11904127 and rs2366264. The combinations of rs11904127/rs2366264/rs62162674 and rs11904127/rs2366264/rs11689667/rs62162674 are the best models to predict cervical cancer.

Our study has some limitations. First, we determined the impact of *TCF7L1* genetic variants on cervical cancer risk; the molecular mechanism of genetic variants on cervical cancer will be conducted in the future. Second, due to limited clinical characteristics obtaining from participates, we will collect more clinical information to detect the association between genetic variants and clinical characteristics in the next work. In spite of the above limitations, our present study provided new candidate biomarkers for the diagnosis of cervical cancer.

## 5. Conclusion

Our study found that *TCF7L1* variations have a protective effect on cervical cancer risk. The results may provide a new perspective on the prevention and diagnosis of cervical cancer.

## Figures and Tables

**Table 1:** Primers used for this study.

SNP_ID	2nd-PCRP	1st-PCRP	UEP DIR	UEP SEQ
rs11904127	ACGTTGGATGTGACTGGCTTCCCCTTGCAT	ACGTTGGATGGTGTGTCCCACAGAGTTCAG	F	TCCCCTTGCATCCCCTG
rs2366264	ACGTTGGATGGCAACAGAGCAAGACAGCAT	ACGTTGGATGGATGGCAATAAATGCTATGG	F	agccAGACAGCATCTCTTTTTTT
rs11689667	ACGTTGGATGGAGGAAGAAATCAATGACCC	ACGTTGGATGAGGAGTACTCACGTAGTCAC	F	aaTTGGAGTTTAATTCAGCGC
rs62162674	ACGTTGGATGTGTTGAACACCTCAACACGG	ACGTTGGATGGGACTAGATGGTCTCCTTTG	F	ccctaAACACGGTCGGCACTCCCA

SNP: single nucleotide polymorphisms; PCRP: polymerase chain reaction primer; UEP-DIR: unextension primer sequence direction; UEP SEQ: unextended mini-sequencing primer sequence. 1st-PCRP means the first PCR primer. 2nd-PCRP means the second PCR primer.

**Table 2:** Characteristic of cervical cancer patients and healthy controls in this study.

Characteristics	Cases (*n* = 508)	Controls (*n* = 497)	*p*
Age, years (mean ± SD)	51.62 ± 9.78	51.36 ± 10.30	0.684
>51	257 (50.6%)	241 (48.5%)	
≤51	251 (49.4%)	256 (51.5%)	
Squamous carcinoma	230 (40.0%)		
Adenocarcinoma	44 (8.7%)		
Missing	234 (51.3%)		
Clinical stage			
I/II	199 (39.2%)		
III/IV	61 (12.0%)		
Missing	164 (48.8%)		

*p* value was calculated by Student's *t*-test. *p* < 0.05 indicates statistical significance.

**Table 3:** Basic information and allele frequencies of *TCF7L1* SNPs.

SNP ID	Chromosome position	Role	Alleles (minor/major)	MAF		O (HET)	E (HET)	*p ^a^*-HWE
				Case	Control			
rs11904127	chr2: 85257695	Intron	A/G	0.364	0.420	0.514	0.487	0.228
rs2366264	chr2: 85261888	Intron	T/G	0.294	0.313	0.428	0.430	0.917
rs11689667	chr2: 85264242	Intron	C/T	0.290	0.316	0.434	0.433	1.000
rs62162674	chr2: 85275113	Intron	C/G	0.297	0.339	0.473	0.448	0.270

SNP: single nucleotide polymorphisms; MAF: minor allele frequency; HWE: Hardy–Weinberg equilibrium. *p* values were calculated by exact test. *p* < 0.05 indicates statistical significance.

**Table 4:** Association analysis between *TCF7L1* SNPs and cervical cancer risk.

SNP ID	Model	Genotype	Case *N*	Control *N*	Without adjusted		With adjusted	
					OR (95% CI)	*p ^a^*	OR (95% CI)	*p ^b^*
rs11904127	Allele	G	644	564			1	
		A	368	408			0.79 (0.66-0.95)	0.010
	Codominant	GG	209	157	1		1	
		GA	226	250	0.68 (0.52-0.89)	0.006	0.68 (0.52-0.89)	0.006
		AA	71	79	0.68 (0.46-0.99)	0.044	0.67 (0.46-0.99)	0.043
	Dominant	GG	209	157	1		1	
		AG-AA	297	329	0.68 (0.52-0.88)	0.003	0.68 (0.52-0.88)	0.003
	Recessive	GG-AG	435	407	1		1	
		AA	71	79	0.84 (0.59-1.19)	0.329	0.84 (0.59-1.19)	0.323
	Log-additive	—	—	—	0.79 (0.66-0.95)	0.010	0.79 (0.66-0.94)	0.010
rs2366264	Allele	G	716	677			1	
		T	298	309			0.91 (0.75-1.10)	0.343
	Codominant	GG	249	233	1		1	
		GT	218	211	0.97 (0.75-1.25)	0.799	0.97 (0.74-1.25)	0.797
		TT	40	49	0.76 (0.49-1.20)	0.245	0.76 (0.48-1.20)	0.244
	Dominant	GG	249	233	1		1	
		TG-TT	258	260	0.93 (0.72-1.19)	0.558	0.93 (0.72-1.19)	0.556
	Recessive	GG-TG	467	444	1		1	
		TT	40	49	0.78 (0.50-1.20)	0.256	0.78 (0.50-1.20)	0.256
	Log-additive	—	—	—	0.91 (0.75-1.10)	0.340	0.91 (0.75-1.10)	0.338
rs11689667	Allele	T	719	674			1	
		C	293	312			0.88 (0.73-1.07)	0.191
	Codominant	TT	253	230	1		1	
		TC	213	214	0.90 (0.70-1.17)	0.452	0.90 (0.70-1.17)	0.447
		CC	40	49	0.74 (0.47-1.17)	0.198	0.74 (0.47-1.17)	0.198
	Dominant	TT	253	230	1		1	
		TC-CC	253	263	0.87 (0.68-1.12)	0.290	0.87 (0.68-1.12)	0.287
	Recessive	TT-TC	466	444	1		1	
		CC	40	49	0.78 (0.50-1.21)	0.260	0.78 (0.50-1.21)	0.261
	Log-additive	—	—	—	0.88 (0.73-1.07)	0.188	0.88 (0.72-1.07)	0.187
rs62162674	Allele	G	711	654			1	
		C	301	336			0.82 (0.68-0.99)	0.044
	Codominant	GG	245	210	1		1	
		GC	221	234	0.81 (0.62-1.05)	0.112	0.81 (0.62-1.05)	0.107
		CC	40	51	0.67 (0.43-1.06)	0.086	0.67 (0.43-1.06)	0.085
	Dominant	GG	245	210	1		1	
		CG-CC	261	285	0.79 (0.61-1.01)	0.057	0.78 (0.61-1.01)	0.054
	Recessive	GG-CG	466	444	1		1	
		CC	40	51	0.75 (0.48-1.15)	0.188	0.75 (0.48-1.15)	0.189
	Log-additive	—	—	—	0.82 (0.67-0.99)	0.039	0.81 (0.67-0.99)	0.038

CI: confidence interval; OR: odds ratio; SNP: single nucleotide polymorphism. *p^a^* values were calculated by logistic regression analysis without adjustment. *p^b^* values were calculated by logistic regression analysis with adjustment for age. *p* < 0.05 indicates statistical significance.

**Table 5:** The association of *TCF7L1* SNPs with the risk of cervical cancer stratified by age.

SNP	Model	Allele/genotype	Case	Control	OR (95% CI)	*p*	Case	Control	OR (95% CI)	*p*
Age			> 51				≤ 51			
rs11904127	Allele	G	327	268	1		317	296	1	
		A	185	206	0.74 (0.57-0.95)	0.019	183	202	0.85 (0.66-1.09)	0.199
	Codominant	GG	108	76	1		101	81	1	
		GA	111	116	0.67 (0.46-0.99)	0.049	115	134	0.69 (0.47-1.01)	0.056
		AA	37	45	0.58 (0.34-0.98)	0.040	34	34	0.80 (0.46-1.40)	0.435
	Dominant	GG	108	76	1		101	81	1	
		AG-AA	148	161	0.65 (0.45-0.94)	0.021	149	168	0.71 (0.49-1.03)	0.068
	Recessive	GG-AG	219	192	1		216	215	1	
		AA	37	45	0.72 (0.45-1.16)	0.173	34	34	0.99 (0.60-1.66)	0.982
	Log-additive	—	—	—	0.74 (0.58-0.96)	0.021	—	—	0.84 (0.64-1.09)	0.186
rs2366264	Allele	G	365	317	1		351	360	1	
		T	147	159	0.80 (0.61-1.05)	0.111	151	150	1.03 (0.79-1.35)	0.816
	Codominant	GG	129	108	1		120	125	1	
		GT	107	101	0.89 (0.61-1.29)	0.531	111	110	1.05 (0.73-1.51)	0.793
		TT	20	29	0.58 (0.31-1.08)	0.083	20	20	1.05 (0.54-2.04)	0.898
	Dominant	GG	129	108	1		120	125	1	
		TG-TT	127	130	0.82 (0.57-1.17)	0.265	131	130	1.05 (0.74-1.49)	0.787
	Recessive	GG-TG	236	209	1		231	235	1	
		TT	20	29	0.61 (0.33-1.11)	0.104	20	20	1.02 (0.53-1.95)	0.950
	Log-additive	—	—	—	0.80 (0.61-1.05)	0.113	—	—	1.03 (0.79-1.36)	0.810
rs11689667	Allele	T	366	315	1		353	359	1	
		C	146	161	0.78 (0.60-1.02)	0.072	147	151	0.99 (0.76-1.30)	0.942
	Codominant	TT	130	106	1		123	124	1	
		TC	106	103	0.84 (0.58-1.22)	0.360	107	111	0.97 (0.67-1.40)	0.873
		CC	20	29	0.56 (0.30-1.05)	0.069	20	20	1.01 (0.52-1.97)	0.975
	Dominant	TT	130	106	1		230	235	1	
		TC-CC	126	132	0.78 (0.55-1.11)	0.165	123	124	0.98 (0.69-1.39)	0.895
	Recessive	TT-TC	236	209	1		127	131	1	
		CC	20	29	0.61 (0.33-1.11)	0.103	20	20	1.03 (0.54-1.96)	0.941
	Log-additive	—	—	—	0.78 (0.60-1.02)	0.073	—	—	0.99 (0.75-1.30)	0.942
rs62162674	Allele	G	362	303	1		349	351	1	
		C	150	175	0.72 (0.55-0.94)	0.014	151	161	0.94 (0.72-1.23)	0.668
	Codominant	GG	126	94	1		119	116	1	
		GC	110	115	0.72 (0.49-1.04)	0.081	111	119	0.91 (0.63-1.31)	0.600
		CC	20	30	0.50 (0.27-0.93)	0.028	20	21	0.93 (0.48-1.81)	0.839
	Dominant	GG	126	94	1		119	116	1	
		CG-CC	130	145	0.67 (0.47-0.96)	0.028	131	140	0.91 (0.64-1.29)	0.602
	Recessive	GG-CG	236	209	1		230	235	1	
		CC	20	30	0.59 (0.32-1.07)	0.080	20	21	0.98 (0.52-1.86)	0.947
	Log-additive	—	—	—	0.71 (0.54-0.93)	0.014	—	—	0.94 (0.71-1.24)	0.659

*p* values were calculated by logistic regression adjusted by age. *p* < 0.05 indicates statistical significance.

**Table 6:** The haplotype frequencies of *TCF7L1* SNPs and their associations with cervical cancer susceptibility.

SNP	Haplotype	Frequency		Without adjusted		With adjusted	
		Case	Control	OR (95% CI)	*p*	OR (95% CI)	*p*
rs2366264|rs11689667|rs62162674	TCC	0.285	0.309	0.89 (0.73-1.08)	0.237	0.89 (0.73-1.08)	0.238
rs2366264|rs11689667|rs62162674	GTC	0.012	0.026	0.44 (0.22-0.88)	0.020	0.43 (0.21-0.86)	0.018
rs2366264|rs11689667|rs62162674	GTG	0.308	0.343	0.84 (0.69-1.02)	0.079	0.84 (0.69-1.02)	0.076

*p* value calculated by Wald test with and without adjusted by age.

**Table 7:** The analysis of SNP-SNP interaction models with the MDR method.

Model	Training Bal. Acc.	Testing Bal. Acc.	CVC	OR (95% CI)	*p*
rs11904127	0.549	0.540	10/10	1.53 (1.18-1.99)	0.001
rs11904127, rs2366264	0.556	0.507	7/10	1.57 (1.21-2.04)	<0.001
rs11904127, rs2366264, rs62162674	0.564	0.507	10/10	1.70 (1.31-2.22)	<0.0001
rs11904127, rs2366264, rs11689667, rs62162674	0.565	0.499	10/10	1.70 (1.31-2.20)	<0.0001

Bal. Acc.: balanced accuracy; CVC: cross-validation consistently. *p* values were calculated by *χ*^2^ test. *p* < 0.05 indicates statistical significance.

## Data Availability

The datasets used during the current study are available from the corresponding author on a reasonable request.

## Supplementary Materials

Supplementary Materials Figure S1: the representative spectra of each SNP in this study. Figure S2: the position of each SNP in the TCF7L1 gene.
