# Supplementary figures and images for: TCF7L1 Genetic Variants Are Associated with the Susceptibility to Cervical Cancer in a Chinese Population
Source: Biomed Res Int. 2021 Mar 20;2021:6670456. doi: 10.1155/2021/6670456 (PMC8007361; doi:10.1155/2021/6670456)

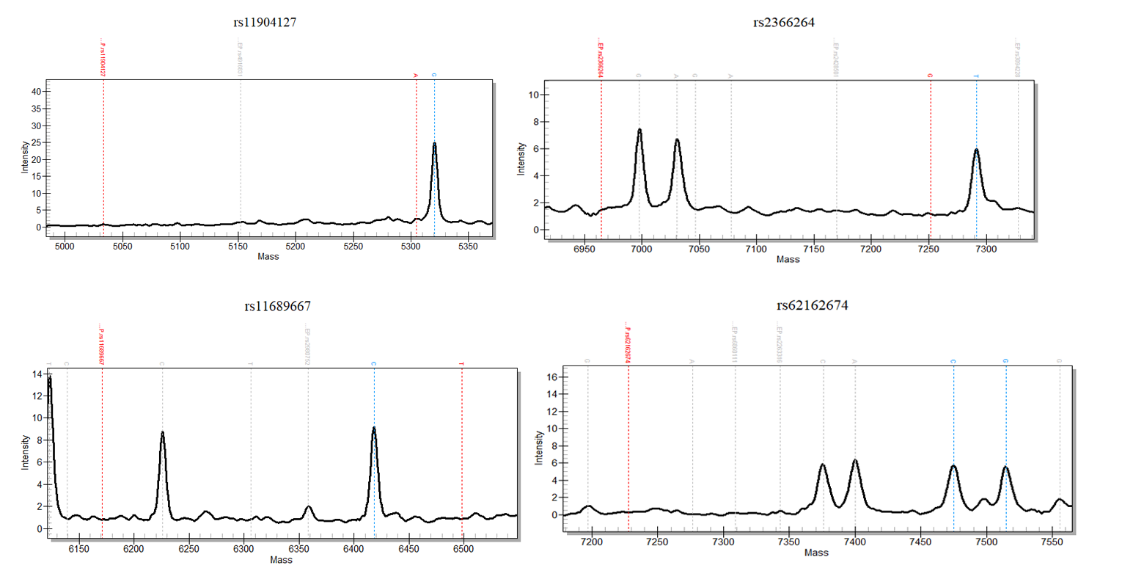

Supplement: Supplementary Materials — Figure S1: the representative spectra of each SNP in this study. Figure S2: the position of each SNP in the TCF7L1 gene. [file 6670456.f1.zip › Fig S1.docx]

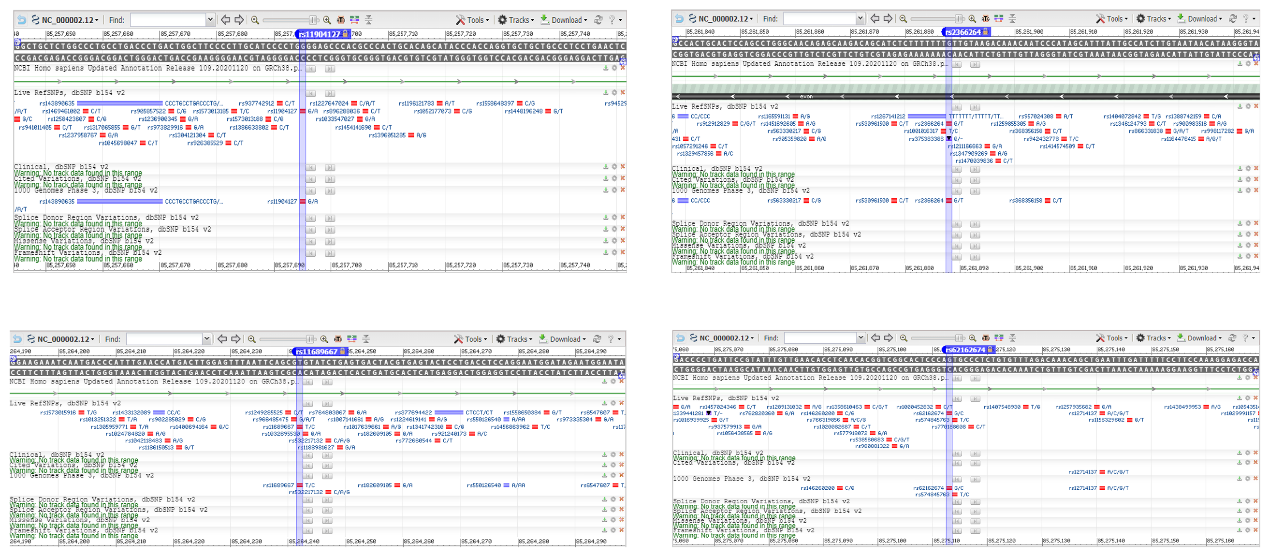

Supplement: Supplementary Materials — Figure S1: the representative spectra of each SNP in this study. Figure S2: the position of each SNP in the TCF7L1 gene. [file 6670456.f1.zip › Fig S2.docx]
